# The genome sequence of the Pygmy False Click Beetle, *Microrhagus pygmaeus* (Fabricius, 1792) (Coleoptera: Eucnemidae)

**DOI:** 10.12688/wellcomeopenres.27729.1

**Published:** 2026-09-07

**Authors:** Michael F. Geiser, Mark G. Telfer, Natural History Museum Genome Acquisition Lab, University of Oxford and Wytham Woods Genome Acquisition Lab, Darwin Tree of Life Barcoding Collective, Wellcome Sanger Institute Tree of Life Management, Samples and Laboratory team, Wellcome Sanger Institute Scientific Operations: Sequencing Operations, Wellcome Sanger Institute Tree of Life Core Informatics team, Tree of Life Core Informatics collective, Darwin Tree of Life Consortium

**Affiliations:** 1 Natural History Museum, London, England, UK; 2 Independent researcher, Ventnor, Isle of Wight, England, UK

**Keywords:** Microrhagus pygmaeus, Pygmy False Click Beetle, genome sequence, chromosomal, Coleoptera

## Abstract

We present a genome assembly from an individual male *Microrhagus pygmaeus* (Pygmy False Click Beetle; Arthropoda; Insecta; Coleoptera; Eucnemidae). The primary assembly has a total length of 320.80 megabases. Most of the primary assembly (91.11%) is scaffolded into 11 chromosomal pseudomolecules, including the X and Y sex chromosomes. The mitochondrial genome has also been assembled, with a length of 35.45 kilobases. This assembly was generated as part of the Darwin Tree of Life project, which produces genomes for eukaryotic species found in Britain and Ireland.

## Species taxonomy

Eukaryota; Opisthokonta; Metazoa; Eumetazoa; Bilateria; Protostomia; Ecdysozoa; Panarthropoda; Arthropoda; Mandibulata; Pancrustacea; Altocrustacea; Allotriocarida; Hexapoda; Insecta; Dicondylia; Pterygota; Neoptera; Eumetabola; Endopterygota; Aparaglossata; Neuropteroidea; Coleoptera; Polyphaga; Elateriformia; Elateroidea; Eucnemidae; *Microrhagus*; *Microrhagus pygmaeus* (Fabricius, 1792) (NCBI:txid445480)

## Background

Microrhagus pygmaeus (Fabricius, 1792), the Pygmy False Click Beetle (Coleoptera: Eucnemidae), is 4–6 mm long ( Brock, 2021). Adults are black to dark brown, with pale hairs on the pronotum and wing cases ( Lompe, 2025). Males have more strongly developed antennae than females ( Jones, 2018).

The species occurs across much of Europe. In Britain it is widely distributed but local, with records concentrated in southern England. It is associated with ancient deciduous woodland and wood pasture, breeding in decaying branches of trees including oak, beech and birch ( Hodge & Yates, 2000). It is listed as Rare (RDB3) in Brock’s 2021 field guide ( Brock, 2021).

Larvae have been recorded developing in a soft, dry hazel twig affected by white rot ( Muona & Teräväinen, 2008). In glass vials, the larvae secreted liquid through specialised areas of the body surface called areoles. This formed a wet path along which they moved from the wood towards moist tissue. The authors suggested that such behaviour could help larvae cross unsuitable areas within decaying wood. Their observations of eucnemid larvae also support the hypothesis that these insects feed mainly on fungal hyphae, releasing digestive fluids outside the body and taking up the resulting liquid ( Muona & Teräväinen, 2008).

We present a chromosome-level genome sequence for *Microrhagus pygmaeus*, produced using the Tree of Life pipeline. The sequence is based on a specimen collected from Ranmore Common, England, UK (Figure 1). This assembly was generated as part of the Darwin Tree of Life Project, which aims to generate high-quality reference genomes for all named eukaryotic species in Britain and Ireland to support research, conservation, and the sustainable use of biodiversity ( Darwin Tree of Life Project Consortium, 2022).

**Figure 1.**
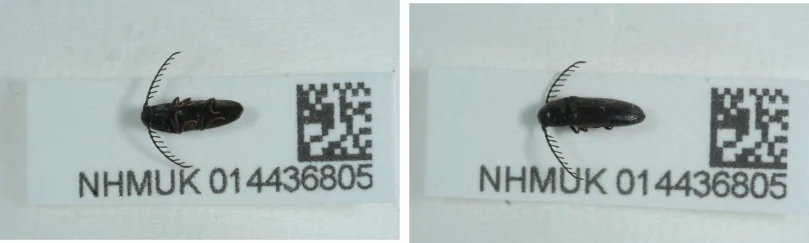
The *M. pygmaeus* specimen (NHMUK014436805, icMicPygm2) used for genome sequencing.

## Methods

### Sample acquisition and DNA barcoding

Two adult male *Microrhagus pygmaeus* specimens were used: the genome-sequencing specimen (specimen ID NHMUK014436805, ToLID icMicPygm2; Figure 1) and the Hi-C specimen (specimen ID Ox001788, ToLID icMicPygm1). The genome-sequencing specimen was collected from Ranmore Common, England, UK (latitude 51.24, longitude −0.39) on 2022-05-21 by Michael Geiser, who also identified it. The Hi-C specimen was collected from Wytham Woods, Oxfordshire, UK (latitude 51.767, longitude −1.345) on 2021-07-08 by Mark Telfer, who also identified it.

The initial identification was verified by an additional DNA barcoding process according to the framework developed by Twyford *et al.*. (2024). A small sample was dissected from the specimen and stored in ethanol, while the remaining parts were shipped on dry ice to the Wellcome Sanger Institute (WSI) (see the protocol). The tissue was lysed, the COI marker region was amplified by PCR, and amplicons were sequenced and compared to the BOLD database, confirming the species identification ( Crowley *et al.* , 2023). Following whole genome sequence generation, the relevant DNA barcode region was also used alongside the initial barcoding data for sample tracking at the WSI ( Twyford *et al.* , 2024). The standard operating procedures for Darwin Tree of Life barcoding are available on protocols.io.

### Nucleic acid extraction

High molecular weight (HMW) DNA extraction protocols developed by the Tree of Life Core Laboratory at the Wellcome Sanger Institute (WSI) are available on protocols.io ( Howard *et al.*, 2025). Tissue from the icMicPygm2 sample was weighed and triaged to select the appropriate extraction workflow. HMW DNA was prepared for one PacBio HiFi library: DTOL14801224. Tissue from the whole organism was homogenised by powermashing using a PowerMasher II tissue disruptor. HMW DNA was extracted using the automated MagAttract v2 workflow. We used centrifuge-mediated fragmentation to produce DNA fragments in the 8–10 kb range, following the Covaris g-TUBE protocol for ultra-low input (ULI). Sheared DNA was purified by automated SPRI (solid-phase reversible immobilisation).

The concentration of the sheared and purified DNA was assessed with a NanoDrop spectrophotometer and a Qubit Fluorometer using the Qubit dsDNA High Sensitivity Assay kit. Fragment size distribution was evaluated by running the sample on the Femto Pulse system. For this sample, final post-shearing DNA measurements included a Qubit concentration of 2.44 ng/μL, and an average fragment size of 10.4 kb.

### PacBio HiFi library preparation and sequencing

Library preparation and sequencing took place in the WSI Scientific Operations core. Library DTOL14801224 was prepared with the recorded ultra-low-input (ULI) PacBio HiFi workflow. Prior to library preparation, DNA for the ultra-low-input preparation was fragmented to approximately 10 kb. The library was prepared with the PacBio SMRTbell® Express Template Prep Kit 2.0 and gDNA Sample Amplification Kit. The sample was normalised to 20 ng DNA. Single-strand overhang removal, DNA damage repair and end-repair/A-tailing were followed by adapter ligation and a 0.85× pre-PCR clean-up with Promega ProNex beads.

The DNA was divided evenly between two PCRs, and each underwent a 0.85× post-PCR clean-up with ProNex beads. DNA concentration was measured with a Qubit Fluorometer v4.0 (Thermo Fisher Scientific) with the Qubit High Sensitivity Assay Kit, and fragment size was assessed using an Agilent Femto Pulse Automated Pulsed Field CE Instrument (Agilent Technologies) with the gDNA 55 kb BAC analysis kit. The two PCRs were pooled to provide at least 500 ng DNA in 47.4 μL. Following DNA damage repair, end-repair/A-tailing and hairpin-adapter ligation, the pooled sample underwent a 1× ProNex bead clean-up. Size selection was performed using a Sage Science PippinHT system, with the target fragment size determined by Femto Pulse analysis. Size-selected libraries were cleaned with 1.0× ProNex beads and normalised to 2 nM before sequencing.

The library was sequenced on a Revio instrument (Pacific Biosciences, California, USA). Prepared libraries with a molarity greater than 2 nM were normalised to 2 nM, and 15 μL was used to make complexes. For libraries with a molarity below 2 nM, the available 10 μL library volume was used without normalisation. Primers were annealed and polymerases were bound to generate circularised complexes following the manufacturer’s instructions. Complexes were purified using 1.2× SMRTbell beads, diluted to a loading concentration of 200–300 pM, and spiked with a Revio sequencing internal control. Sequencing used Revio 25M SMRT cells. SMRT Link software was used to configure and monitor the run and to perform primary and secondary data analysis.

### Hi-C

***Sample preparation and crosslinking***

Hi-C data were generated from the icMicPygm1 whole organism sample using the Arima-HiC v2 kit (Arima Genomics). Following the manufacturer’s instructions, tissue was fixed and DNA crosslinked using TC buffer to a final formaldehyde concentration of 2%. The tissue was homogenised using the Diagnocine Power Masher-II. Crosslinked DNA was digested with a restriction enzyme master mix, biotinylated, and ligated. Clean-up was performed with SPRISelect beads before library preparation. DNA concentration was measured with the Qubit Fluorometer (Thermo Fisher Scientific) and Qubit HS Assay Kit. The biotinylation percentage was estimated using the Arima-HiC v2 QC beads.

***Hi-C library preparation and sequencing***

Biotinylated DNA constructs were fragmented using a Covaris E220 sonicator and size-selected to 400–600 bp using SPRISelect beads. DNA was enriched with Arima-HiC v2 kit Enrichment beads. End repair, A-tailing, and adapter ligation were carried out with the NEBNext Ultra II DNA Library Prep Kit (New England Biolabs), following a modified protocol where library preparation occurs while DNA remains bound to the Enrichment beads. Libraries were amplified with KAPA HiFi HotStart mix and a custom Unique Dual Index (UDI) barcode set (Integrated DNA Technologies). Depending on sample concentration and biotinylation percentage determined at the crosslinking stage, libraries were amplified with 10–16 PCR cycles. Post-PCR clean-up was performed with SPRISelect beads. Libraries were quantified using the AccuClear Ultra High Sensitivity dsDNA Standards Assay Kit (Biotium) and a FLUOstar Omega plate reader (BMG Labtech).

Prior to sequencing, libraries were normalised to 10 ng/μL. Normalised libraries were quantified again to create equimolar and/or weighted 2.8 nM pools. Pool concentrations were checked using the Agilent 4200 TapeStation (Agilent) with High Sensitivity D500 reagents before sequencing. Libraries were sequenced on the Illumina NovaSeq 6000, generating paired-end 150-bp reads.

### Genome assembly

Prior to assembly of the PacBio HiFi reads, a database of *k*-mer counts ( *k* = 31) was generated from the filtered reads using FastK. The *k*-mer frequency distribution was analysed with GenomeScope (version 2.1.0) ( Ranallo-Benavidez *et al.*, 2020), providing fitted point estimates of genome size, heterozygosity and repeat content.

The HiFi reads were assembled using Hifiasm ( Cheng *et al.*, 2021) with the --primary option. The Hi-C reads ( Rao *et al.* , 2014) were mapped to contigs using bwa-mem2 ( Vasimuddin *et al.*, 2019), and the contigs were scaffolded in YaHS ( Zhou *et al.*, 2023) with the --break option for handling potential misassemblies. The scaffolded assemblies were evaluated using Gfastats ( Formenti *et al.* , 2022), BUSCO ( Manni *et al.*, 2021) and MerquryFK ( Rhie *et al.*, 2020).

The mitochondrial genome was assembled using MitoHiFi ( Uliano-Silva *et al.* , 2023) and OATK ( Zhou *et al.*, 2025).

### Assembly curation

The assembly was decontaminated using the Assembly Screen for Cobionts and Contaminants ( ASCC) pipeline. The flat files and maps for curation were generated with TreeVal. Manual curation was conducted primarily in PretextView and HiGlass ( Kerpedjiev *et al.* , 2018). Scaffolds were visually inspected and corrected as described by Howe *et al*. (2021). Manual corrections included 125 breaks and 151 joins. This reduced the scaffold count by 23.1%, increased the scaffold N50 by 112.5%, and reduced the total assembly length by 0.9%. The curation process is described at sanger-tol/curation-resources. A Hi-C contact map of the primary assembly was generated with PretextSnapshot.

### Assembly quality assessment

The MerquryFK tool ( Rhie *et al.*, 2020) was run in a Singularity container ( Kurtzer *et al.*, 2017) to evaluate *k*-mer completeness and assembly quality for the primary and alternate haplotypes using the *k*-mer database ( *k* = 31) computed prior to genome assembly. The analysis outputs included assembly QV scores and completeness statistics.

The primary assembly was assessed using BlobToolKit ( Challis *et al.*, 2020). PacBio reads were mapped to the primary assembly to derive sequence coverage, and BUSCO ( Manni *et al.*, 2021) was used to assess gene-set completeness. DIAMOND BLASTp searches of extracted BUSCO proteins and DIAMOND BLASTx searches of assembly sequences against UniProt Reference Proteomes ( Buchfink *et al.*, 2021), followed by BLASTn searches of sequences without DIAMOND BLASTx hits against the NCBI nt database ( Altschul *et al.*, 1990), supported taxonomic assignment. Coverage, sequence composition, taxonomic assignment and BUSCO results were integrated into a BlobDir for interactive inspection and visualisation. The snail and blob plots were rendered locally from this hosted BlobDir using BlobTk v0.8.3. The scaffold-length radial scale function for the snail plot was set to linear ( Challis & Blaxter, 2026). The blob plot used circular markers with area proportional to scaffold length ( Challis *et al.*, 2020).

Ancestral linkage groups were painted along the chromosome-level scaffolds. Complete BUSCO genes (single-copy and duplicated) from the coleoptera_odb12 lineage dataset were assigned to the 8 Coleoptera ancestral linkage groups (C1–C7 and CX) described by Maulana *et al.*. (2026) and plotted along chromosomes drawn to scale.

## Genome sequence report

### Sequence data

PacBio sequencing of the *M. pygmaeus* specimen generated 83.68 Gb (gigabases) from 9.79 million reads. We assembled the genome from these reads. GenomeScope analysis using a *p* = 2 model gave a *k*-mer-based haploid genome size estimate of 271.91 Mb, with heterozygosity of 0.41% and repeat content of 43.56%; the full-model fit was 92.62% (Figure 2). The GenomeScope estimates guided expectations for the assembly. The sequencing data provided approximately 274× coverage of the primary assembly. Hi-C sequencing produced 114.57 Gb from 379.38 million reads, which were used to scaffold the primary assembly. Table 1 summarises the specimen and sequencing details.

**Figure 2.**
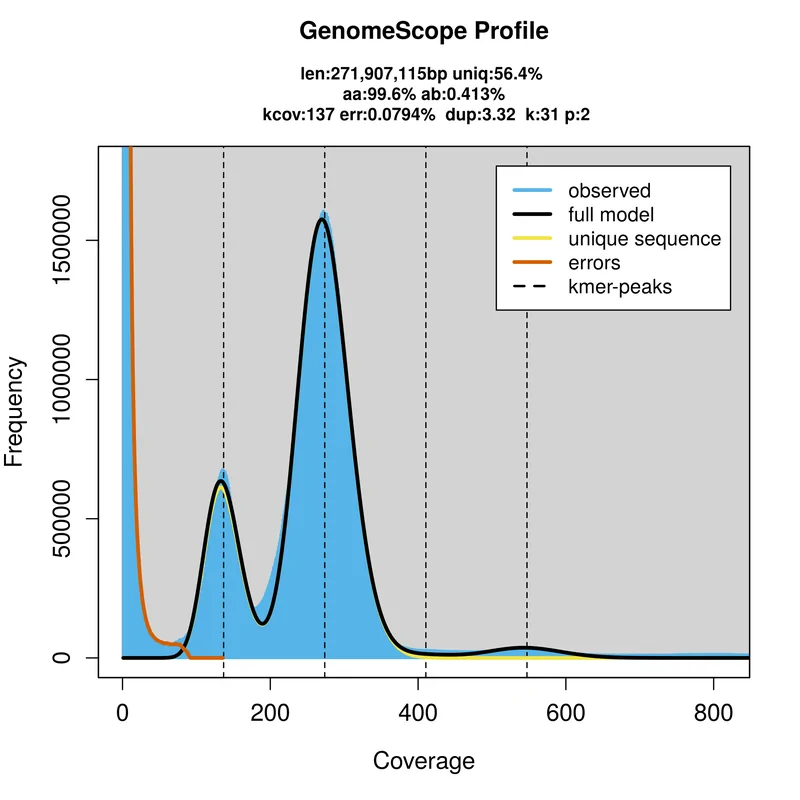
GenomeScope model fitted to the *k*-mer frequency distribution. The plot shows the observed and modelled *k*-mer spectra used to estimate genome size, heterozygosity and repeat content from unassembled sequencing reads.

**Table 1.** Specimen and sequencing data for *M. pygmaeus* (BioProject PRJEB86941)

**Platform**	**PacBio HiFi**	**Hi-C**
**ToLID**	icMicPygm2	icMicPygm1
**Specimen ID**	NHMUK014436805	Ox001788
**BioSample (source individual)**	SAMEA114805832	SAMEA10979047
**BioSample (tissue)**	SAMEA114806001	SAMEA10979437
**Tissue**	whole organism	whole organism
**Instrument**	Revio (1 run)	Illumina NovaSeq 6000
**Library ID & run accession(s)**	DTOL14801224: ERR14749979	ERR14749207
**Read count total**	9.79 million reads	379.38 million read pairs
**Base count total**	83.68 Gb	114.57 Gb

### Assembly statistics

The primary assembly was generated and an alternate haplotype assembly was also deposited at scaffold level in INSDC databases. The primary assembly is proposed as the reference assembly for this species. It has a total length of 320.80 Mb in 195 scaffolds (excluding organelle sequences), with 361 gaps, and a scaffold N50 of 28.48 Mb (Table 2). The primary assembly has a scaffold auN of 26.4 Mb.

**Table 2.** Genome assembly statistics for *M. pygmaeus*

**Genome assembly**	**Primary assembly**	**Alternate haplotype assembly**
**Assembly name**	icMicPygm2.1	icMicPygm2.1 alternate haplotype
**Assembly accession**	GCA_965643785.1	GCA_965643775.1
**Assembly level**	chromosome	scaffold
**Span (Mb)**	320.80	445.27
**Number of chromosomes**	11	-
**Number of contigs**	556	16,560
**Contig N50 (Mb)**	1.27	0.04
**Number of scaffolds (excluding organelle sequences)**	195	16,551
**Scaffold N50 (Mb)**	28.48	0.04
**Scaffold auN (Mb)**	26.4	-
**Longest scaffold length (Mb)**	34.39	1.31
**Sex chromosomes**	X and Y	-
**Organelles**	Mitochondrial genome: 35.45 kb	-

Most of the primary assembly sequence (91.11%) was assigned to 11 chromosome-level scaffolds, representing 9 autosomes and the X and Y sex chromosomes. These chromosome-level scaffolds, confirmed by Hi-C data, are named according to size (Figure 3; Table 3). The X and Y chromosomes were assigned based on read coverage. During genome curation, we noted that the exact order and orientation of the contigs are uncertain in the following regions: chromosome 1 (13,712–15,573 Kbp), chromosome 2 (13,291–14,760 Kbp), chromosome 3 (15,994–19,128 Kbp), chromosome 4 (12,850–13,937 Kbp), chromosome 5 (13,575–15,445 Kbp), chromosome 6 (11,312–15,191 Kbp), chromosome 7 (12,027–13,300 Kbp), chromosome 8 (14,397–15,671 Kbp), chromosome 9 (10,470–12,272 Kbp), and chromosome X (13,700–15,044 Kbp). The Hi-C signal suggests a heterozygous reciprocal translocation between Chromosome 4 (28,521–30,059 Kbp) and Chromosome 9 (0–5,386 Kbp). We noted that a large number of centromeric repeats is in the unlocalised sequences.

The mitochondrial genome was also assembled (length 35.45 kb, OZ286117.1). This sequence is included as a contig in the multifasta file of the primary genome submission and as a standalone record.

**Figure 3.**
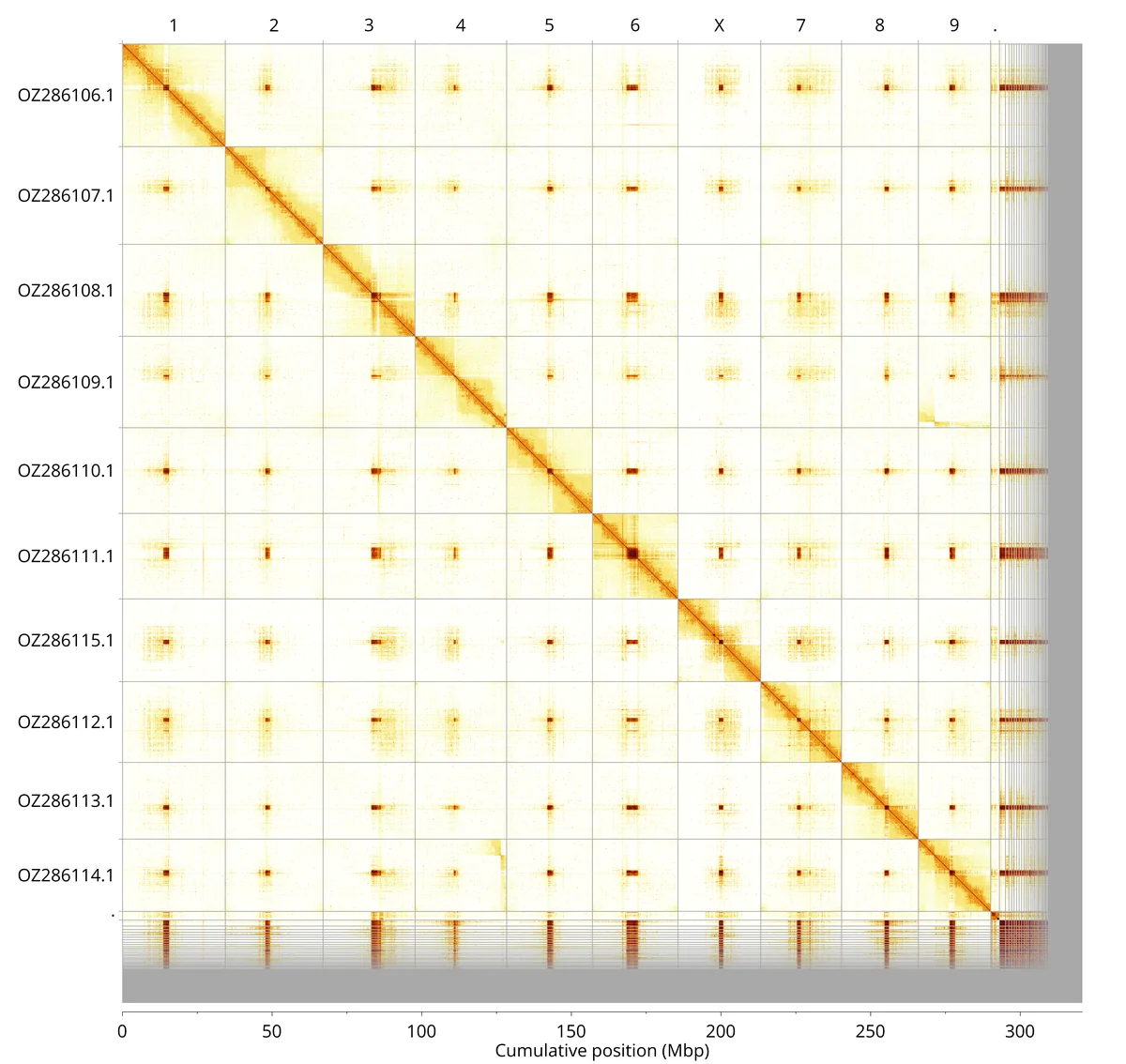
Hi-C contact map of the *M. pygmaeus* primary assembly. Assembled chromosomes are shown in order of size and labelled along the axes, with a megabase scale shown below. The plot was generated using PretextSnapshot.

**Table 3.** Chromosomal pseudomolecules in the primary genome assembly of *M. pygmaeus* icMicPygm2

**INSDC accession**	**Molecule**	**Length (Mb)**	**GC%**
OZ286106.1	1	34.39	36.50
OZ286107.1	2	32.55	36.50
OZ286108.1	3	30.67	37.50
OZ286109.1	4	30.47	36
OZ286110.1	5	28.57	36.50
OZ286111.1	6	28.48	36.50
OZ286112.1	7	26.90	37.50
OZ286113.1	8	25.60	36
OZ286114.1	9	24.08	36.50
OZ286115.1	X	27.61	36
OZ286116.1	Y	2.98	38.50

Chromosome painting shows the distribution of Coleoptera ancestral linkage groups assignments across the chromosome-level scaffolds (Figure 4).

**Figure 4.**
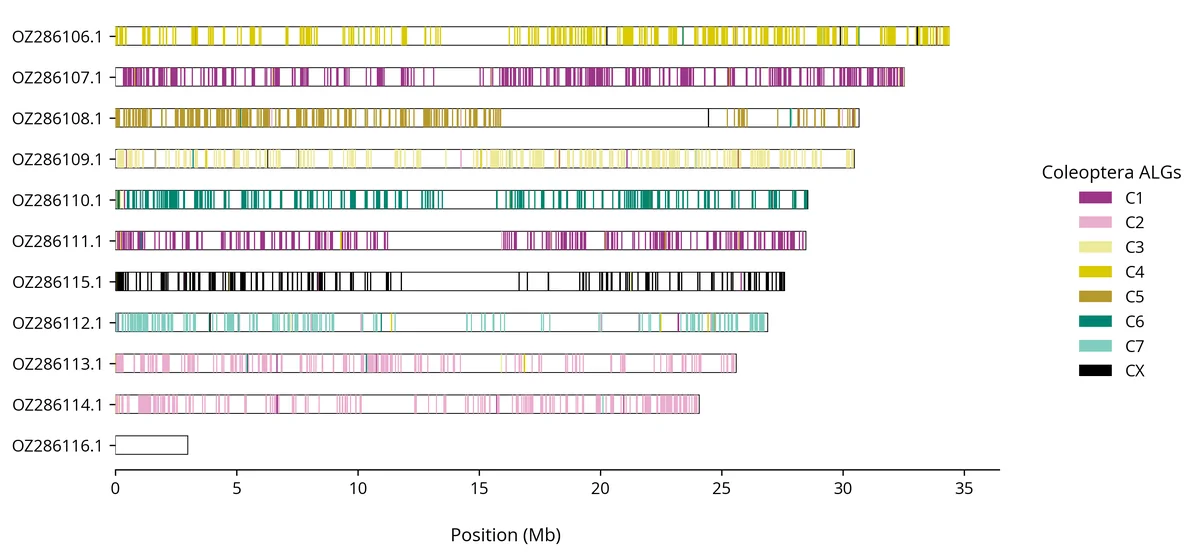
Coleoptera ancestral linkage groups painting of the genome assembly of *M. pygmaeus*. Chromosomes are drawn to scale, with coloured bars marking the positions of complete coleoptera_odb12 BUSCO genes. Each BUSCO gene is coloured by its assignment to one of the 8 Coleoptera ALGs (C1–C7 and CX).

### Assembly quality metrics

The combined primary and alternate assemblies achieve an estimated QV of 55.4. The *k*-mer completeness is 93.45% for the primary assembly, 76.21% for the alternate haplotype, and 98.27% for the combined assemblies (Figure 5).

**Figure 5.**
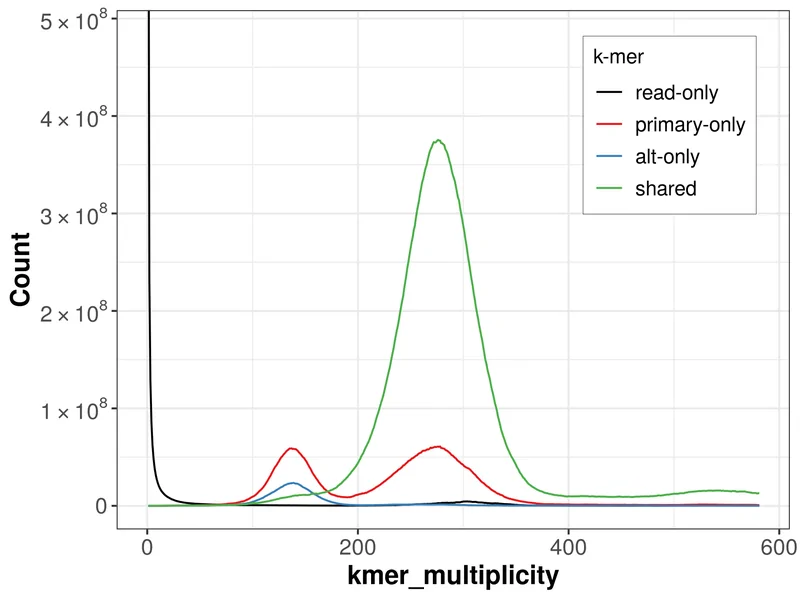
Evaluation of *k*-mer completeness using MerquryFK. This plot illustrates the recovery of *k*‐mers from the original read data in the primary assembly, the alternate haplotype assembly and the combined assemblies. The horizontal axis represents *k*‐mer multiplicity, and the vertical axis shows the number of *k*‐mers. The black curve represents *k*‐mers that appear in the reads but are not assembled. The green curve corresponds to *k*‐mers shared by both haplotypes, and the red and blue curves show *k*‐mers found only in one of the haplotypes.

BUSCO v.5.8.3 analysis using the endopterygota_odb10 reference set ( *n* = 2,124) identified 97.9% of the expected gene set (single = 97.5%, duplicated = 0.4%).

The snail plot in Figure 6 provides a summary of assembly contiguity, cumulative scaffold count, sequence composition and BUSCO completeness for the primary assembly ( Challis & Blaxter, 2026). The relative sizes of the longest scaffold, scaffold N50 and N90, together with the dark-grey scaffold-length distribution, show how much of the assembly is contained in long scaffolds. The outer composition track shows variation in GC, AT and N content, while the central track shows the cumulative scaffold count and the BUSCO inset summarises gene-set completeness.

The blob plot in Figure 7 displays scaffolds from the primary assembly by GC proportion and base coverage. Marker area is proportional to scaffold length, and colour indicates taxonomic assignment ( Challis *et al.*, 2020).

**Figure 6.**
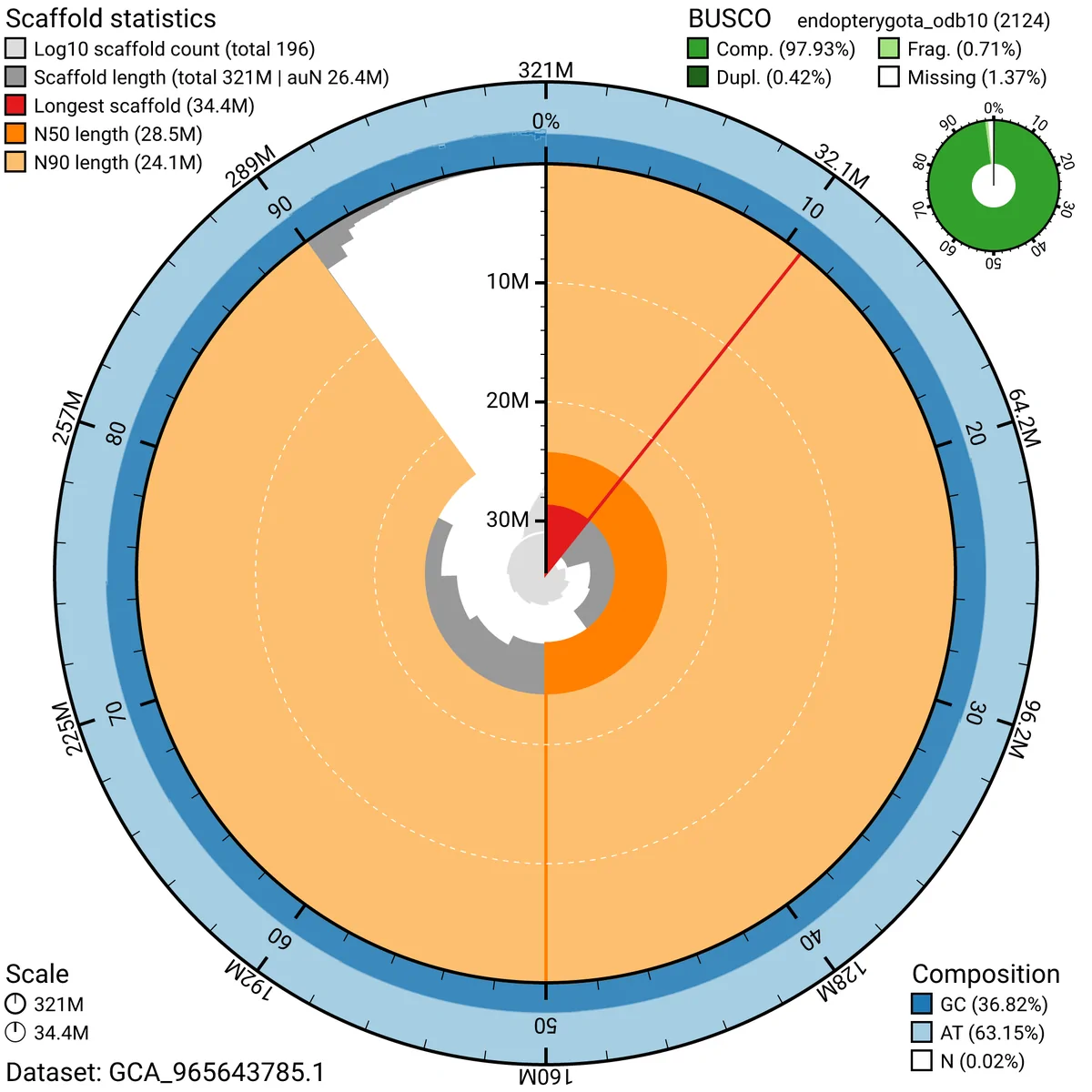
Assembly metrics for icMicPygm2.1, the primary assembly. The BlobToolKit snail plot provides an overview of assembly metrics and BUSCO gene completeness. The circumference represents the length of the primary assembly, and the main plot is divided into 1,000 bins around the circumference. The outermost blue tracks display the distribution of GC, AT, and N percentages across the bins. Scaffolds are arranged clockwise from longest to shortest and are depicted in dark grey. Scaffold length is shown on a linear radial scale, scaled to the longest scaffold. Red shading indicates the longest scaffold, and the deeper orange and pale orange shading represent the N50 and N90 lengths. The summary statistics include scaffold auN, the area under the scaffold Nx curve. The light grey spiral at the centre shows the cumulative scaffold count on a logarithmic scale. A summary of complete, fragmented, duplicated, and missing BUSCO genes in the endopterygota_odb10 set is presented at the top right. The assembly can be explored interactively in the BlobToolKit viewer.

**Figure 7.**
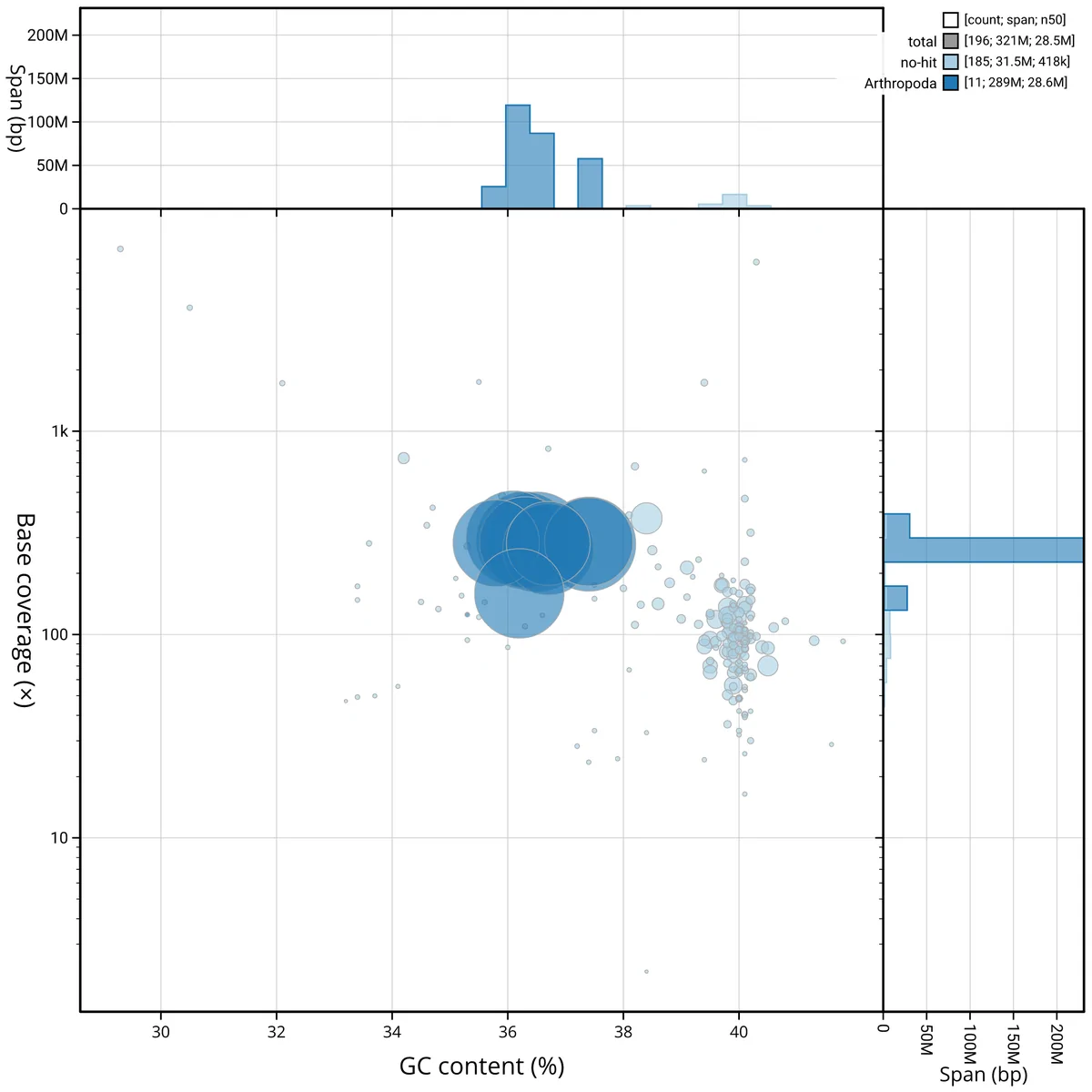
BlobToolKit blob plot for icMicPygm2.1. The plot shows base coverage (vertical axis) and GC content (horizontal axis). Circles represent scaffolds; marker area is proportional to scaffold length, and colour indicates taxonomic assignment. The histograms along the axes display the total length of sequences distributed across different levels of coverage and GC content. The assembly can be explored interactively in the BlobToolKit viewer.

Table 4 lists the assembly metric benchmarks adapted from Rhie *et al.*. (2021) and the Earth BioGenome Project (EBP) Report on Assembly Standards. The three-part EBP metric, calculated for the primary assembly, is **6.C.Q61**.

**Table 4.** Earth BioGenome Project summary metrics for the *M. pygmaeus* assembly

**Measure**	**Value**	***Benchmark***
EBP summary (primary)	6.C.Q61	*6.C.Q40*
Contig N50 length	1.27 Mb	*> 1 Mb*
Scaffold N50 length	28.48 Mb	*= chromosome N50*
Consensus quality (QV)	Primary: 61.6; alternate: 54.4; combined: 55.4	*≥ 40*
*k*-mer completeness	Primary: 93.45%; alternate: 76.21%; combined: 98.27%	*> 90%*
BUSCO	C:97.9% [S:97.5%, D:0.4%], F:0.7%, M:1.4%, n:2,124	*S > 90%; D < 5%*
Percentage of assembly assigned to chromosomes	91.11%	*≥ 90%*

**Notes:** The EBP summary uses log10(Contig N50); chromosome-level (C) or log10(Scaffold N50); Q (Merqury QV). BUSCO v5.8.3 using the endopterygota_odb10 lineage dataset: C=complete; S=single-copy; D=duplicated; F=fragmented; M=missing; n=orthologues.

### Data availability

European Nucleotide Archive: Microrhagus pygmaeus. Accession number PRJEB86941; https://identifiers.org/ena.embl/PRJEB86941. The genome sequence is released openly for reuse. The *M. pygmaeus* genome sequencing initiative is part of the Darwin Tree of Life Project ( PRJEB40665) and Sanger Institute Tree of Life Programme ( PRJEB43745). All raw sequence data and the primary and alternate assemblies have been deposited in INSDC databases. The genome will be annotated using available RNA-Seq data and presented through the Ensembl pipeline at the European Bioinformatics Institute. Raw data and assembly accession identifiers are reported in Table 1 and Table 2.

Production code used in genome assembly at the WSI Tree of Life is available at https://github.com/sanger-tol. Table 5 lists software versions used in this study.

**Table 5.** Software versions and sources used for *M. pygmaeus*

**Software**	**Version**	**Source**
BLAST	2.14.0	https://ftp.ncbi.nlm.nih.gov/blast/executables/blast+/
BlobToolKit	4.4.6	https://github.com/genomehubs/blobtoolkit
BlobTk (hosted BlobDir)	0.5.1	https://github.com/genomehubs/blobtk
BlobTk (snail and blob plot renderer)	0.8.3	https://github.com/genomehubs/blobtk
BUSCO	5.8.3; 6.1.0	https://gitlab.com/ezlab/busco
busco-alg-painter	0.1.0	https://github.com/Karenvn/busco-alg-painter
bwa-mem2	2.2.1	https://github.com/bwa-mem2/bwa-mem2
DIAMOND	2.1.8	https://github.com/bbuchfink/diamond
fasta_windows	0.2.4	https://github.com/tolkit/fasta_windows
FastK	1.2	https://github.com/thegenemyers/FASTK
GenomeScope	2.1.0	https://github.com/tbenavi1/genomescope2.0
Gfastats	1.3.6	https://github.com/vgl-hub/gfastats
Hifiasm	0.19.8-r603	https://github.com/chhylp123/hifiasm
HiGlass	1.13.4	https://github.com/higlass/higlass
MerquryFK	1.1.0-c1	https://github.com/thegenemyers/MERQURY.FK
Minimap2	2.28-r1209	https://github.com/lh3/minimap2
MitoHiFi	3.2.2	https://github.com/marcelauliano/MitoHiFi
Oatk	1.0	https://github.com/c-zhou/oatk
MultiQC	1.14; 1.17 and 1.18	https://github.com/MultiQC/MultiQC
Nextflow	24.10.4	https://github.com/nextflow-io/nextflow
PretextSnapshot	0.0.6	https://github.com/sanger-tol/PretextSnapshot
PretextView	1.0.3	https://github.com/sanger-tol/PretextView
samtools	1.21	https://github.com/samtools/samtools
sanger-tol/ascc	0.1.0	https://github.com/sanger-tol/ascc
sanger-tol/blobtoolkit	v0.8.0	https://github.com/sanger-tol/blobtoolkit
sanger-tol/curationpretext	1.4.2	https://github.com/sanger-tol/curationpretext
Seqtk	1.4-r122	https://github.com/lh3/seqtk
Singularity	3.9.0	https://github.com/sylabs/singularity
TreeVal	1.2.2	https://github.com/sanger-tol/treeval
YaHS	1.2.2	https://github.com/c-zhou/yahs

### Author information

Contributors are listed at the following links:

- Members of the University of Oxford and Wytham Woods Genome Acquisition Lab
- Members of the Natural History Museum Genome Acquisition Lab
- Members of the Darwin Tree of Life Barcoding collective
- Members of the Wellcome Sanger Institute Tree of Life Management, Samples and Laboratory team
- Members of Wellcome Sanger Institute Scientific Operations – Sequencing Operations
- Members of the Wellcome Sanger Institute Tree of Life Core Informatics team
- Members of the Tree of Life Core Informatics collective
- Members of the Darwin Tree of Life Consortium

### Wellcome Sanger Institute – Legal and Governance

The materials that have contributed to this genome note have been supplied by a Darwin Tree of Life Partner. The submission of materials by a Darwin Tree of Life Partner is subject to the **‘Darwin Tree of Life Project Sampling Code of Practice’**, which can be found in full on the Darwin Tree of Life website. By agreeing with and signing up to the Sampling Code of Practice, the Darwin Tree of Life Partner agrees they will meet the legal and ethical requirements and standards set out within this document in respect of all samples acquired for, and supplied to, the Darwin Tree of Life Project. Further, the Wellcome Sanger Institute employs a process whereby due diligence is carried out proportionate to the nature of the materials themselves, and the circumstances under which they have been/are to be collected and provided for use. The purpose of this is to address and mitigate any potential legal and/or ethical implications of receipt and use of the materials as part of the research project, and to ensure that in doing so we align with best practice wherever possible. The overarching areas of consideration are:

- Ethical review of provenance and sourcing of the material
- Legality of collection, transfer and use (national and international)

Each transfer of samples is further undertaken according to a Research Collaboration Agreement or Material Transfer Agreement entered into by the Darwin Tree of Life Partner, Genome Research Limited (operating as the Wellcome Sanger Institute), and in some circumstances, other Darwin Tree of Life collaborators.
