# Supplementary material for: The genome sequence of the Pygmy False Click Beetle, Microrhagus pygmaeus (Fabricius, 1792) (Coleoptera: Eucnemidae)
Source: Wellcome Open Res. 2026 Sep 7;11:606. [Version 1] doi: 10.12688/wellcomeopenres.27729.1 (PMC13646543; doi:10.12688/wellcomeopenres.27729.1)
Supplement: Peer review history

# Reviewer report: Reviewer response for version 1

**DOI:** 10.21956/wellcomeopenres.30520.r171567

**Published:** 2026-10-12

**Authors:** Laurent Falquet

**Affiliations:** 1 University of Fribourg, Fribourg, Switzerland

The authors present the complete genome of the insect Microrhagus pygmaeus.

 The work is extremely well detailed, the assembly quality has been assessed in great depth. I would like to congratulate the authors for this excellent manuscript.

 There are a couple a improvements that could have been added.

 For example, the authors do not explain why they performed a "primary" assembly and not assembled the two phased haplotigs. Given the high quality datasets (PacBio HiFi and HiC), this phasing would have been possible. 

 Other possible improvement, the authors didn't check for telomeres completeness on the assembled chromosomes.

 If this organism lost its telomeric sequence motif, then it should be stated in the text.

 Finally I am not sure to understand why Figure 1 shows two pictures of the same individual (NHMUK014436805, ToLID icMicPygm2). Shouldn't the second specimen ID Ox001788 ToLID icMicPygm1 be shown side by side?

 Also I would have appreciated in the "Data availability" section the COI markers and accession numbers in the BOLD database of the two specimen (example DTNHM6543-23.COI-5P for NHMUK014436805).

Are the datasets clearly presented in a usable and accessible format, and the assembly and annotation available in an appropriate subject-specific repository?

Yes

Are sufficient details of the sequencing and extraction, software used, and materials provided to allow replication by others?

Yes

Are the rationale for sequencing the genome and the species significance clearly described?

Yes

Are the protocols appropriate and is the work technically sound?

Yes

Reviewer Expertise:

Metagenomics and Bioinformatics

I confirm that I have read this submission and believe that I have an appropriate level of expertise to confirm that it is of an acceptable scientific standard.

# Reviewer report: Reviewer response for version 1

**DOI:** 10.21956/wellcomeopenres.30520.r170037

**Published:** 2026-10-08

**Authors:** Jhon Alex Dziechciarz Vidal

**Affiliations:** 1 Universidade Federal de SãoCarlos (UFSCar), São Carlos, Brazil

 Review of “The genome sequence of the Pygmy False Click Beetle, *Microrhagus pygmaeus* (Fabricius, 1792) (Coleoptera: Eucnemidae).”

 The manuscript presents the genome assembly of *Microrhagus pygmaeus*, a beetle species distributed across Britain. The assembly is of high quality, with all chromosomes identified, including the X and Y sex chromosomes. The assembly metrics are strong, supported by high sequencing depth for both HiFi and Hi-C data, high BUSCO completeness, and favourable Merqury metrics. The analysis of ancestral linkage groups suggests a high degree of chromosomal conservation, with no apparent chromosomal rearrangements detected.

 Despite the overall quality of the manuscript, I have several comments and suggestions.

1. Some species names are not italicised. Please ensure that scientific names are consistently italicised throughout the manuscript.
2. The authors state that the X and Y chromosomes were identified using sequencing coverage analysis, but no plot is provided to support this conclusion. Please include a coverage plot showing the evidence used to identify the sex chromosomes.
3. Based on my experience with invertebrate genome reports, particularly those involving beetles, I suggest that the consortium consider including higher-resolution photographs, using additional specimens if necessary. Figure 1 does not provide sufficient morphological detail to facilitate independent identification by other taxonomists. Given the complexity of coleopteran taxonomy and the morphological similarity among many species, detailed photographs of the head, body, antennae, and aedeagus, where applicable, would help document diagnostic morphological characters and complement the COI sequence data.
4. Hi-C data reflect chromosomal organisation, which may vary among individuals. I recognise that obtaining both HiFi and Hi-C data from the same individual may be challenging in such a small species. However, could the authors explain why the specimen used for Hi-C sequencing was not collected from the same population as the specimen used for genome sequencing?
5. Although we are now in the genomic era, biological specimens and cytogenetic data remain highly valuable. I encourage the authors and the consortium to consider obtaining cytogenetic data for the species whose genomes they assemble, ideally from the sequenced individuals or specimens from the same population. These data could provide independent validation of chromosome number and sex chromosome constitution, thereby increasing confidence in the assemblies. This is particularly relevant because some beetle species possess multiple sex chromosomes or B chromosomes.
6. Although the genome assembly has already been deposited, I suggest that, for future assemblies, the authors consider adopting a consistent chromosome orientation, with the centromere positioned closer to the beginning of each chromosome sequence where appropriate. The centromeric regions were not identified in this study, but the Hi-C map in Figure 3 may provide clues to putative centromeric locations. These locations would require independent validation before being used to guide chromosome orientation.

Are the datasets clearly presented in a usable and accessible format, and the assembly and annotation available in an appropriate subject-specific repository?

Yes

Are sufficient details of the sequencing and extraction, software used, and materials provided to allow replication by others?

Yes

Are the rationale for sequencing the genome and the species significance clearly described?

Yes

Are the protocols appropriate and is the work technically sound?

Yes

Reviewer Expertise:

Cytogenetics, Cytogenomics, Genome Assembly.

I confirm that I have read this submission and believe that I have an appropriate level of expertise to confirm that it is of an acceptable scientific standard.

# Reviewer report: Reviewer response for version 1

**Published:** 2026-09-07

**Authors:** Automated Benchmarking

**Affiliations:** 1 Wellcome Sanger Institute, Hinxton, UK

As this article is a Genome Note, Automated Benchmarking has been used to determine whether the reported assembly passes three defined checks established by the Earth BioGenome Project: the raw and assembled data are publicly available, the Earth BioGenome Project three-part metric is met, and the BUSCO score passes the stated threshold. For Microrhagus pygmaeus (icMicPygm2), all three checks passed. Data available: pass. ENA study PRJEB86941, raw-read accessions ERR14749979 and ERR14749207, and assembly accessions GCA_965643785.1 and GCA_965643775.1 are public. Earth BioGenome Project three-part metric: pass. The primary assembly is reported as 6.C.Q61, meeting the 6.C.Q40 reference standard. BUSCO: pass. The primary assembly, which is proposed as the reference assembly for this species, has 97.9% complete BUSCOs (97.5% single-copy and 0.4% duplicated). It meets the target of greater than 90% complete and single-copy BUSCOs. This decision uses values reported in the Genome Note and public accession records.

Reviewer Expertise:

NA

I confirm that I have read this submission and believe that I have an appropriate level of expertise to confirm that it is of an acceptable scientific standard.
